# The role of frontline autologous stem cell transplantation for primary plasma cell leukemia: a retrospective multicenter study (KMM160)

**DOI:** 10.18632/oncotarget.18535

**Published:** 2017-06-16

**Authors:** Sung-Hoon Jung, Je-Jung Lee, Kihyun Kim, Cheolwon Suh, Dok Hyun Yoon, Chang-Ki Min, Sang Kyun Sohn, Chul Won Choi, Ho Sup Lee, Hyo Jung Kim, Ho-Jin Shin, Soo-Mee Bang, Sung-Soo Yoon, Seong Kyu Park, Ho-Young Yhim, Min Kyoung Kim, Jae-Cheol Jo, Yeung-Chul Mun, Jae Hoon Lee, Jin Seok Kim, Korean Multiple Myeloma Working Party

**Affiliations:** ^1^ Chonnam National University Hwasun Hospital, Hwasun, Jeollanamdo, Republic of Korea; ^2^ Samsung Medical Center, Sungkyunkwan University School of Medicine, Seoul, Republic of Korea; ^3^ Asan Medical Center, University of Ulsan College of Medicine, Seoul, Republic of Korea; ^4^ Seoul St. Mary’s Hospital, The Catholic University of Korea, Seoul, Republic of Korea; ^5^ Kyungpook National University Hospital, Daegu, Republic of Korea; ^6^ Korea University School of Medicine, Seoul, Republic of Korea; ^7^ Kosin University Gospel Hospital, Busan, Republic of Korea; ^8^ Hallym University Sacred Heart Hospital, Anyang, Republic of Korea; ^9^ Pusan National University Hospital, Busan, Republic of Korea; ^10^ Seoul National University Bundang Hospital, Seoul, Republic of Korea; ^11^ Seoul National University Hospital, Seoul, Republic of Korea; ^12^ Soonchunhyang University Bucheon Hospital, Bucheon, Republic of Korea; ^13^ Chonbuk National University Medical School, Jeonju, Republic of Korea; ^14^ Yeungnam University Medical Center, Daegu, Republic of Korea; ^15^ Ulsan University Hospital, Ulsan, Republic of Korea; ^16^ Ewha Womans University School of Medicine, Seoul, Republic of Korea; ^17^ Gachon University Gil Medical Center, Incheon, Republic of Korea; ^18^ Severance Hospital, Yonsei University College of Medicine, Seoul, Republic of Korea

**Keywords:** primary plasma cell leukemia, treatment, autologous stem cell transplantation, prognosis

## Abstract

Primary plasma cell leukemia (pPCL) is a rare and aggressive plasma cell neoplasm, with rapidly progressing clinical course. We evaluated the treatment status and survival outcomes of 69 Korean patients with pPCL. Of them, 59 patients were treated; 15 (25.4%) were treated initially with novel agent-based regimens with upfront autologous stem cell transplantation (ASCT), 7 (11.9%) with conventional chemotherapy with upfront ASCT, 21 (35.6%) with novel agent-based regimens only, and 16 (27.1%) were treated with conventional chemotherapy alone. Overall response rates after initial therapy were significantly higher in patients treated with novel agent-based regimens compared with those treated with conventional chemotherapies (75% vs. 43.4%, *P =* 0.026). Median progression-free survival (PFS) and overall survival (OS) were 12.2 months and 16.1 months, respectively. The median PFS of the four treatment groups–conventional chemotherapy alone, novel agents alone, conventional chemotherapy with ASCT, and novel agents with ASCT–were 1.2, 9.0, 10.5, and 26.4 months, respectively (*P* < 0.001); the median OS of the four treatment groups were 2.9, 12.3, 14.1, and 31.1 months, respectively (*P* < 0.001). The median OS was also significantly better in the patients with novel agents with ASCT versus other patients. In a multivariate analysis, an increased lactate dehydrogenase level, low albumin (< 3.5 g/dL), and non-CR after front-line treatment were independently associated with poor PFS and OS. In conclusion, the use of novel agent-based therapy with ASCT and achieving a deep response to front-line treatment are important in expecting improved PFS and OS in patients with pPCL.

## INTRODUCTION

Plasma cell leukemia (PCL) is a rare and highly aggressive plasma cell dyscrasia that occurs in 1–4% of patients with multiple myeloma (MM) [1, 2]. It is defined by the presence of more than 20% plasma cells in peripheral blood and an absolute plasma cell count ≥ 2 × 10^9^/L [3, 4]. PCL is classified as primary and secondary; ‘primary’ is used when PCL presents in patients without previous evidence of MM. Primary PCL (pPCL) represents ∼ 60% of PCL, and has distinct clinical and laboratory features at presentation compared with those of MM. pPCL is generally diagnosed at a younger age than MM or secondary PCL. Patients with pPCL have more advanced International Staging System (ISS) stage of disease at presentation, frequently accompanied by hepatomegaly, splenomegaly, extramedullary disease, and extensive bone disease. Additionally, renal failure, hypercalcemia, thrombocytopenia, increased lactate dehydrogenase (LDH), and elevated plasma cell labeling index are more frequent in pPCL than MM [4, 5]. In studies investigating pPCL, it has been shown that pPCL has a different biological feature than MM in terms of immunophenotype expression, cytogenetics, gene-expression profiles, and microRNA signatures. Multiparametric flow cytometry revealed that PCL shows higher expression of CD20 antigens and lower CD9, CD56, CD117, and HLA-DR than MM [6, 7]. The frequencies of t(4;14), t(11;14), and t(14;16) were higher in pPCL, and del(17p13.1) was detected in about 13–50% of pPCL cases [8–11]. In a study investigating gene expression profiles in 21 patients with pPCL, a 503-gene signature, especially related to the NFκB pathway, structural organization of the cell, and cell adhesion/migration differed from that in MM [12].

With these unfavorable clinical and biological characteristics at presentation, patients with pPCL have a dismal prognosis, with a median overall survival (OS) of only 7–12 months with conventional chemotherapy alone [1, 6, 13]. Although a few recent studies have suggested that the use of novel agents and performing autologous stem cell transplantation (ASCT) increased the survival outcomes in pPCL [14–16], the prognosis is still poor compared with MM, and the optimal therapy remains undefined.

In this study, we collected data on Korean patients with pPCL. We then analyzed the clinical and cytogenetic characteristics, survival outcomes, and prognostic factors associated with OS.

## RESULTS

### Patient population

In total, 69 (0.6%) patients were diagnosed with pPCL among the 12,347 patients with MM from 18 institutions between February 1998 and December 2015. Of them, 65 (94.2%) patients were diagnosed since 2005; the incidence was not different between the two time periods. Baseline clinical characteristics of all patients are summarized in Table 1. The median age of the patients was 63 years (range, 34–85 years); 46.4% were 65 years or older. The most common heavy-chain isotype was IgG (58.0%); 29.0% of patients had light chain disease. Thirty-three (47.8%) patients had low serum albumin levels (< 3.5 g/dL) and 48 (69.6%) patients had high ß_2_-microglobulin levels (> 5.5 mg/L). Regarding the ISS, 3 (4.3%) patients were stage I, 16 (23.2%) were stage II, and 48 (69.6%) were stage III. Thirty-nine (56.5%) patients showed leukocytosis (≥ 11 ×10^9^/L), and 78.3% of patients had a platelet value of < 130 × 10^9^/L at diagnosis. Organomegaly was detected in 23 (33.3%) patients and extramedullary disease was seen in 13 (18.8%) patients. Osteolytic bone lesions were present in 42 (60.9%) patients. Results of conventional cytogenetic studies and FISH were available in 46 and 42 patients, respectively. Results were mostly abnormal. The most common abnormalities on conventional cytogenetic studies were complex (63.0%) and hypodiploid (34.8%). Del(13q12), del(17p13), and t(11;14) were found in 13, 11, and 10 patients, respectively (Table 2).

**Table 1:** Baseline clinical characteristics of all patients (*n* = 69)

Variables	
Median age, year (range) ≥ 65, *n*. (%)	63.0 (34–85) 32 (46.4)
Male, *n*. (%)	42 (60.9%)
ECOG PS ≥ 2	38 (55.1%)
Immunoglobulin (Ig) type, *n*. (%) IgG IgA IgM Light chain only	40 (58.0) 8 (11.6) 1 (1.4) 20 (29.0)
International Staging System, *n* (%) I II III	3 (4.3) 16 (23.2) 48 (69.6)
Serum hemoglobin, median (g/dL)	8.1 (4.1–13.1)
White blood cell count, median (×10^9^/L)	13.0 (1.9–64.7)
Platelet count, median (×10^9^/L)	90.0 (10–248)
Serum creatinine ≥ 2 mg/dL	29 (42.0%)
LDH > 1 × ULN	42 (60.9%)
Serum ß_2_-microglobulin, median (mg/L) > 5.5 mg/L, *n* (%)	8.9 (4.6–86.9) 48 (69.6%)
Serum albumin, median (g/dL)	3.5 (1.9–4.9)
Serum calcium, median (mg/dL)	9.4 (6.2–15.3)
Organomegaly	23 (33.3%)
Lytic bone lesions	42 (60.9%)
Extramedullary involvement	13 (18.8%)

Abbreviations: *n*, number; ECOG, Eastern Cooperative Oncology Group; PS, performance status; LDH, lactate dehydrogenase; ULN, upper limit of normal value.

**Table 2:** Cytogenetic abnormalities in the patients with primary plasma cell leukemia (*n* = 53)

Cytogenetic abnormalities	Total number (*n =* 53)	conventional cytogenetic studies (*n =* 46)	fluorescent *in situ* hybridization (*n =* 42)
Complex	29	29	-
Hypodiploid	16	16	-
del(13q14)	13	12	12
del(17p13)	11	5	9
t(11;14)	10	9	6
t(4;14)	9	1	8
t(14;16)	5	1	4
1q rearrangement	6	3	3
Not evaluable	16		

### Treatments

Of the 69 patients, 59 were treated. The frontline treatments and responses are summarized in Table 3. Of the treated patients, 36 (61.0%) received a novel agent-based regimen as the first-line regimen, such as bortezomib, cyclophosphamide, and dexamethasone (VCD), or bortezomib, melphalan, and prednisolone (VMP), or bortezomib, doxorubicin, and dexamethasone (PAD), or cyclophosphamide, thalidomide, dexamethasone (CTD), or thalidomide and dexamethasone (TD), or bortezomib, thalidomide, and dexamethasone (VTD). Another 23 (39.0%) patients were treated with conventional chemotherapy as a first-line regimen such as, vincristine, doxorubicin, and dexamethasone (VAD), or melphalan and prednisolone, or cyclophosphamide and dexamethasone, or high dose dexamethasone, or cytarabine alone, or cyclophosphamide, doxorubicin, vincristine, and dexamethasone. Of the 23 patients who were treated with conventional chemotherapy as the first-line regimen, 12 eventually received a novel agent-based regimen as a salvage therapy. Thus, 48 (69.6%) patients received novel-agent-based regimes during the treatment period.

**Table 3:** Summary of response according to front-line treatments (*n* = 59)

Patients undergoing upfront ASCT (*n =* 22)							
		Best response after induction therapy			Best response after ASCT		
Front-line regimen, *n* (%)		CR	VGPR	≥ PR	CR	VGPR	≥ PR
Novel agent-based	15 (68.2%)	4 (26.7%)	4 (26.7%)	12 (80.0%)	7 (46.7%)	3 (20.0%)	13 (86.7%)
Bortezomib-based	4	1	2	4	2	1	4
Thalidomide-based	11	3	2	8	5	2	9
Bortezomib+thalidomide	0	0	0	0	0	0	0
Conventional chemotherapy	7 (31.8%)	2 (28.6%)	2 (28.6%)	6 (85.7%)	5 (71.4%)	0	5 (71.4%)
Patients receiving chemotherapy alone (*n =* 37)							
Novel agent-based	21 (56.7%)	4 (19.0%)	3 (14.3%)	15 (71.4%)			
Bortezomib-based	15	2	3	10			
Thalidomide-based	3	1	0	2			
Bortezomib+thalidomide	3	1	0	3			
Conventional chemotherapy	16 (43.2%)	0	0	4 (25.0%)			

Abbreviations: *n*, number; ASCT, autologous stem cell transplantation; CR, complete response; VGPR, very good partial response; PR, partial response.

Overall response rates (ORR) after initial therapy were significantly higher in patients treated with novel agent-based regimens compared with those treated with conventional chemotherapies (75% vs. 43.4%, *P =* 0.026). In patients who were treated with novel agent-based regimens, there was no difference of ORR between bortezomib- and thalidomide-based regimens. Upfront ASCT was performed in 22 (37.3%) patients. Twenty-one patients maintained the best response at ASCT and one patient who had achieved VGPR after conventional chemotherapy eventually lost the response and progressed before ASCT. Twelve (54.5%) patients achieved complete remission (CR) after ASCT. One patient received consolidation therapy with bortezomib and dexamethasone after ASCT and 10 received maintenance therapy with thalidomide after ASCT. One (1.7%) patient received the allogeneic stem cell transplantation (SCT) after initial VMP induction chemotherapy, and progressed at 9.3 months after allogeneic SCT.

### Survival outcomes and factors associated OS

**Figure 1:**
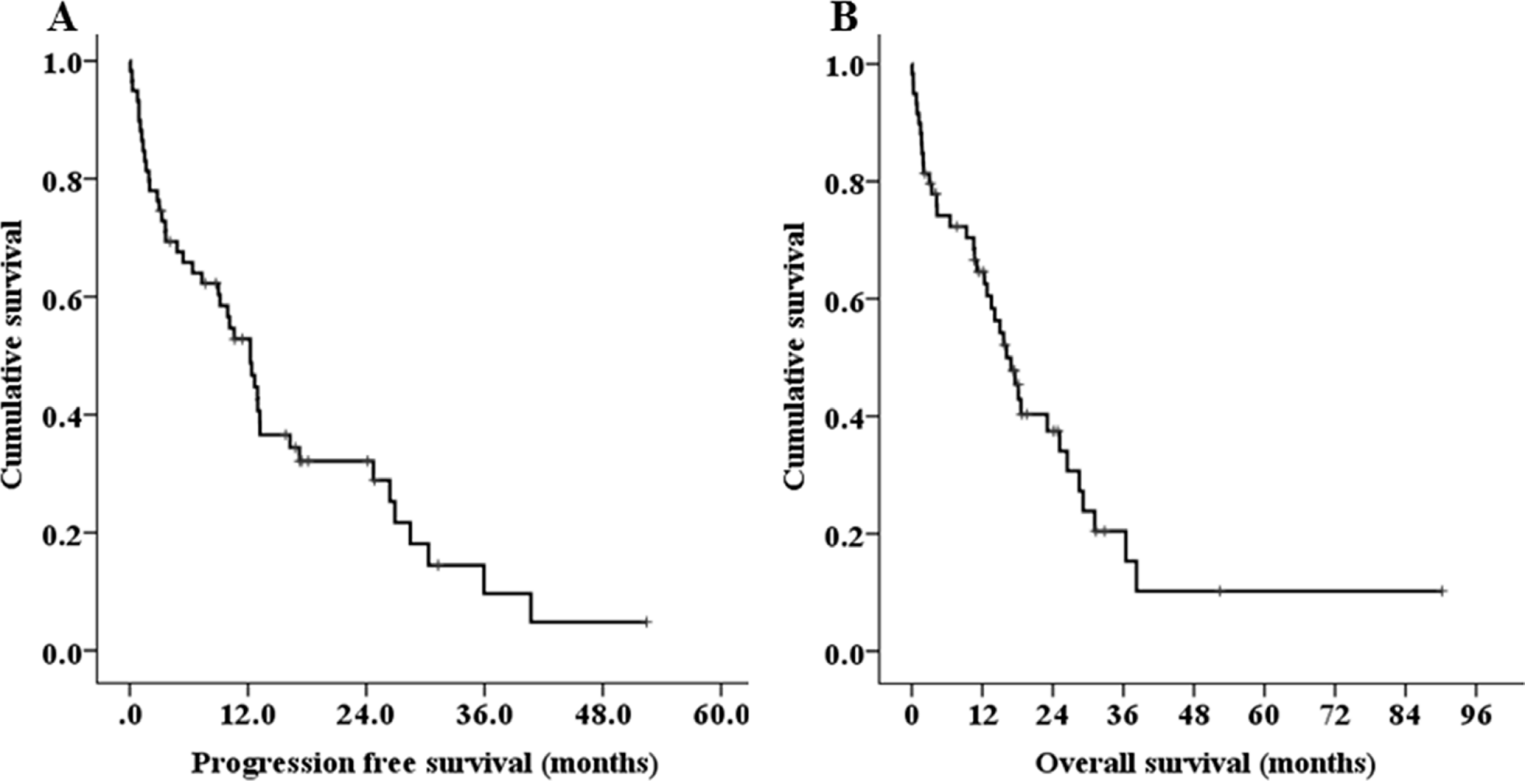
Kaplan-Meier survival curves for progression-free survival (PFS) (**A**) and overall survival (OS) (**B**) of treated patients (*n* = 59).

**Figure 2:**
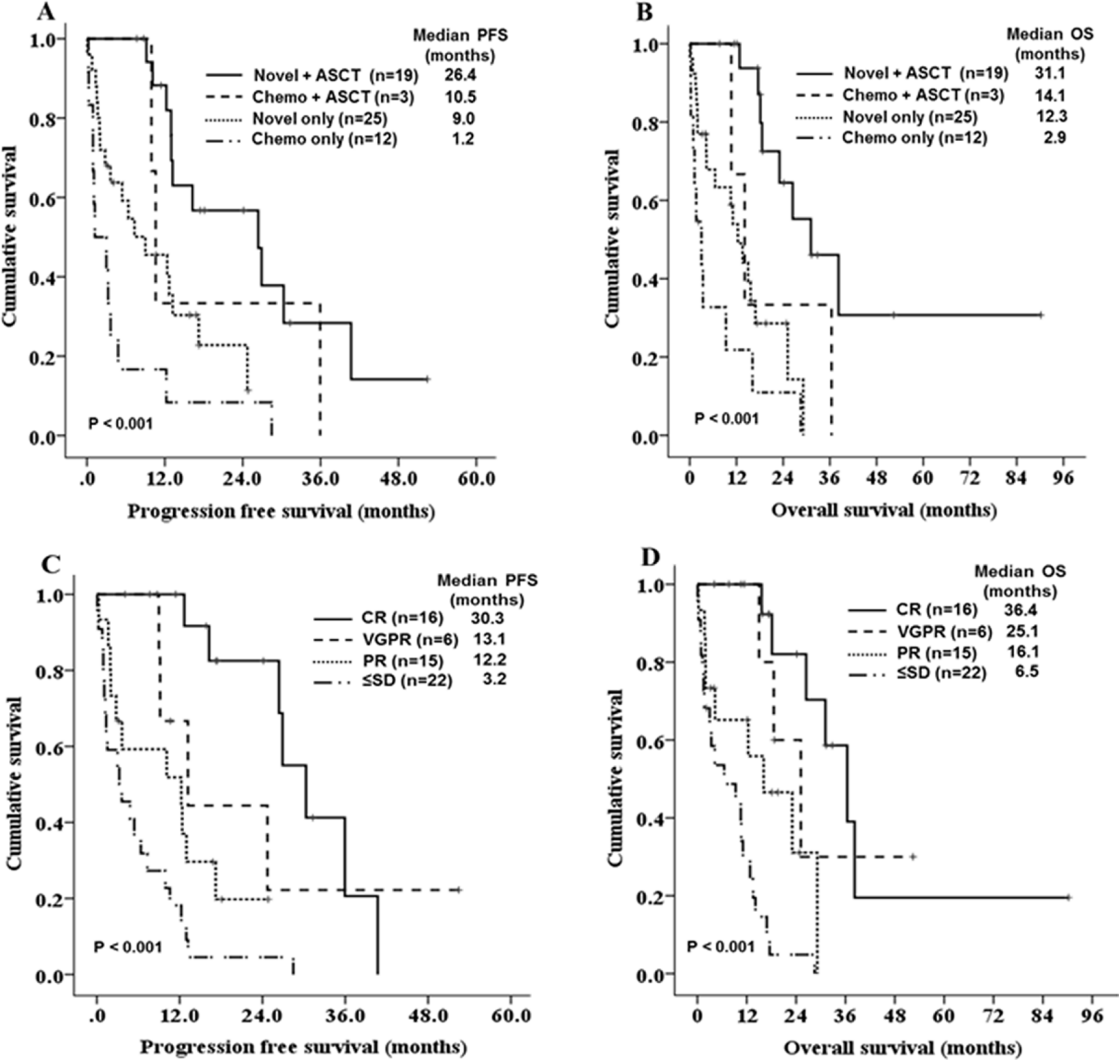
Kaplan-Meier survival curves for PFS and OS according to treatment groups (**A, B**) and best response to front-line treatment (**C, D**).

Over a median follow-up of 16.5 months, the median PFS and OS were 12.2 months (95% CI 9.2–15.2, Figure 1A), and 16.1 months (95% CI 12.0–20.2, Figure 1B). In total, 5 (8.5%) patients died within less than 1 month and 20 (33.9%) died within less than 1 year following the diagnosis [early mortality(EM)]. The major causes of early mortality were disease progression and infection. The EM rate was significantly lower in patients who were initially treated with novel agents compared with those treated with conventional chemotherapies (22.2% vs. 52.5%, HR 0.262, 95% CI 0.084–0.814, *P =* 0.025). Patients who were initially treated with novel-agents had increased PFS and OS compared with those who were initially treated with conventional chemotherapy, but the difference was not statistically significant (PFS: 12.9 vs. 4.8 months, *P =* 0.059, OS: 18.5 vs. 10.6 months, *P =* 0.168). The median PFS of the four treatment groups–conventional chemotherapy alone, novel agents alone, conventional chemotherapy with ASCT, and novel agents with ASCT–were 1.2, 9.0, 10.5, and 26.4 months, respectively (*P <* 0.001, Figure 2A). The median OS was also significantly better in patients who were treated with novel agents with ASCT (2.9 months in conventional chemotherapy alone vs. 12.3 months in novel agents alone vs. 14.1 months in conventional chemotherapy with ASCT vs. 31.1 months in novel agents with ASCT, *P <* 0.001, Figure 2B). In young patients (< 65 years), the median OS for patients undergoing ASCT was significantly longer than those who did not undergo ASCT (31.1 vs. 2.9 months, *P <* 0.001). Regarding the outcomes according to best response to front-line therapy, the median PFS and OS were significantly better in patients achieving a CR compared with those with very good partial responses, partial responses, or stable disease/progressive disease (*P <* 0.001 for both, Figure 2C, 2D).

**Figure 3:**
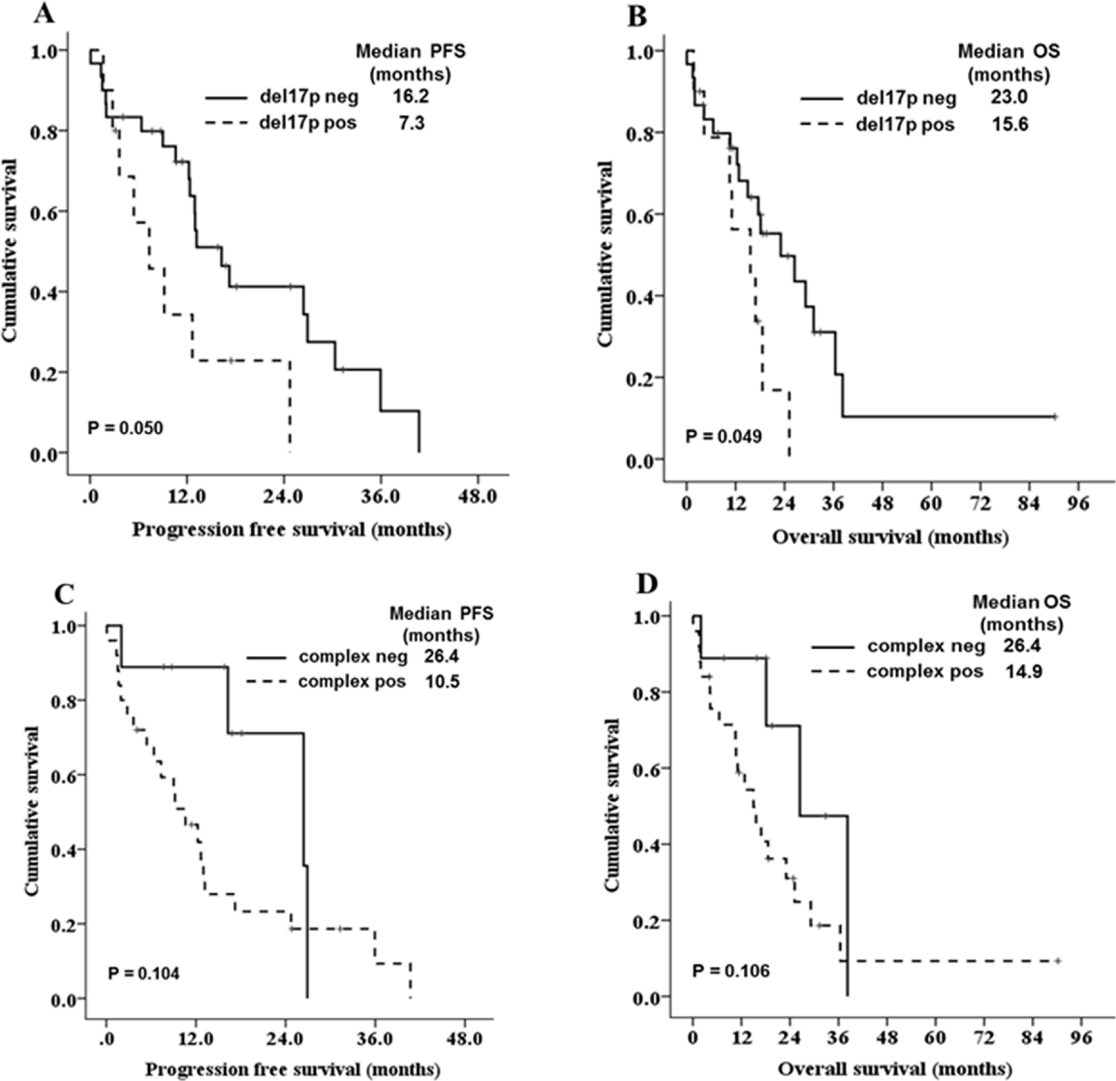
Kaplan-Meier survival curves for PFS and OS according to del(17p13) (**A, B**) and complex karyotype (**C, D**) in patients (*n* = 47) except those who received conventional chemotherapy alone.

We evaluated the factors associated with PFS and OS in the 59 patients who were treated. The univariate analysis demonstrated that five clinical factors were associated with PFS and OS (*P <* 0.1); these factors were increased LDH level, thrombocytopenia, low serum albumin level, high serum β2-microglobulin and non-CR after front-line treatment. Additionally, del17p was associated with OS in univariate analysis (Table 4). Cox multivariate analysis for PFS and OS was performed. Increased LDH level, low serum albumin, and non-CR after front-line treatment were associated significantly with PFS and OS (Table 5). The prognostic impact of each cytogenetic abnormality was evaluated in univariate analyses. Although patients with del17p showed a trend towards shorter OS compared to those without del17p (15.6 vs. 18.1 months, respectively, *P =* 0.086), none showed significant prognostic value. In a subgroup analysis (*n =* 47) excluding those patients who received conventional chemotherapy alone, patients with del17p had significantly shorter PFS and OS compared to those without del17p (Figure 3A, 3B). Additionally, patients with complex karyotypes showed a trend towards poor survival outcomes compared to those without complex karyotypes (Figure 3C, 3D).

**Table 4:** Univariate analysis of risk factors for progression-free survival and overall survival (*n* = 59)

Variables	PFS		OS	
	HR (95% CI)	*P*-value	HR (95% CI)	*P*-value
Age > 65 years	1.51 (0.80–2.84)	0.196	1.64 (0.83–3.21)	0.147
Gender	0.61 (0.33–1.14)	0.127	0.69 (0.36–1.34)	0.282
PS ECOG ≥ 2	1.47 (0.80–2.70)	0.204	1.69 (0.89–3.21)	0.109
Organomegaly	1.39 (0.73–2.66)	0.310	1.28 (0.64–2.52)	0.477
Extramedullary disease	0.76 (0.36–1.61)	0.483	1.06 (0.50–2.26)	0.870
Leukocytosis (≥ 11 × 10^9^/L)	1.28 (0.68–2.39)	0.432	1.16 (0.61–2.22)	0.644
LDH > (1 × ULN)	4.74 (2.16–10.41)	< 0.001	6.32 (2.44–16.35)	< 0.001
Platelets < 130 × 10^9^/L	2.17 (0.90–5.22)	0.083	2.18 (0.90–5.28)	0.084
Hemoglobin < 8.0 g/dL	0.62 (0.33–1.17)	0.143	0.63 (0.32–1.22)	0.178
Serum creatinine ≥ 2 mg/dL	1.36 (0.71–2.62)	0.345	1.43 (0.73–2.78)	0.286
Serum calcium > 11.0 mg/dl	1.65 (0.85–3.20)	0.137	1.63 (0.82–3.22)	0.160
Serum albumin < 3.5 g/dL	2.02 (1.09–3.74)	0.025	1.96 (1.02–3.75)	0.042
Serum β2-microglobulin > 5.5 mg/L	2.16 (1.01–4.60)	0.045	2.12 (0.98–4.55)	0.054
BM plasma cells > 60%	1.36 (0.72–2.57)	0.337	0.55 (0.27–1.12)	0.100
Complex karyotype	1.64 (0.72–3.70)	0.233	1.82 (0.77–4.28)	0.168
del(17p13)	1.84 (0.84–4.03)	0.125	2.03 (0.90–4.55)	0.086
Non-CR after front-line treatment	4.68 (2.03–10.76)	< 0.001	5.10 (2.06–12.63)	< 0.001

Abbreviations: ECOG, Eastern Cooperative Oncology Group; PS, performance status; LDH, lactate dehydrogenase; ULN, upper limit of normal value; BM, bone marrow; CR, complete response; ASCT, autologous stem cell transplant.

**Table 5:** Multivariate analysis of risk factors for progression-free survival and overall survival (*n* = 59)

	Progression-free survival	
	Hazard ratio (95% CI)	*P*-value
LDH > (1 × ULN)	6.21 (2.04–16.02)	< 0.001
Non-CR after front-line treatment	4.23 (1.55–11.51)	0.005
Serum albumin < 3.5 g/dL	2.69 (1.40–5.17)	0.003
Platelets < 130 × 10^9^/L	0.93 (0.35–2.48)	0.893
Serum β2-microglobulin > 5.5 mg/L	1.81 (0.77–4.24)	0.173
	**Overall survival**	
	**Hazard ratio (95% CI)**	***P*-Value**
LDH > (1 × ULN)	8.03 (2.11–30.48)	0.002
Non-CR after front-line treatment	8.31 (2.20–31.33)	0.002
Serum albumin < 3.5 g/dL	3.25 (1.42–7.42)	0.005
Serum β2-microglobulin > 5.5 mg/L	2.59 (0.97–6.91)	0.058
Platelets < 130 × 10^9^/L	1.42 (0.37–5.35)	0.604
del(17p13)	0.88 (0.35–2.18)	0.788

Abbreviations: LDH, lactate dehydrogenase; ULN, upper limit of normal value; CR, complete response; ASCT, autologous stem cell transplant.

## DISCUSSION

Over the last two decades, the development of more effective agents and increased use of ASCT have resulted in improvements in survival outcome in patients with MM. However, this improvement did not clearly translate to patients with pPCL. The optimal induction therapy for pPCL patients is still unclear and survival outcome is insufficient compared with MM. A few retrospective studies have shown that treatment with novel agents induced higher response rates and prolonged survival than conventional chemotherapies. The Italian GIMEMA Multiple Myeloma Working Party reported a retrospective study of 29 patients with pPCL who were treated with frontline therapy with bortezomib. A bortezomib-based regimen was effective with an ORR of 79%, including ≥ VGPR (very good partial response) in 38% [17]. Additionally, a prospective study with lenalidomide and dexamethasone for pPCL resulted in an ORR of 73.9%, with at least 39% VGPR in 23 patients with pPCL [18]. In the present study, induction therapy with novel agents decreased the EM rate and increased the response rates compared with conventional therapy. Bortezomib- or thalidomide-based regimens achieved ORRs of ∼70%, and combination treatment with bortezomib and thalidomide achieved more than PR in all patients. In a report of two cases with secondary PCL, combined therapy with bortezomib, lenalidomide, and dexamethasone resulted in CR [19]. Thus, a combination of novel agents such as bortezomib and thalidomide, or bortezomib and lenalidomide, seems to be very promising as a front-line therapy for pPCL.

Although frontline therapy with novel agents decreased the EM rates and increased the response rate in our study, it did not significantly improve the survival in patients with pPCL. The most improved survival was achieved in patients who received treatment with novel agents incorporated with ASCT. There are limited data on the role of transplantation in pPCL. The largest reported study is a retrospective analysis of 272 patients who underwent ASCT by the European Group for Blood and Marrow Transplantation [20]. A higher proportion of patients with pPCL than MM patients were in CR at day 100 post-transplantation (41.2% vs. 28.2%, *P <* 0.001), but median PFS and OS were significantly shorter compared with MM (PFS: 14.3 vs. 27.4 months, OS: 25.7 vs. 62.2 months). In a study of 97 patients with pPCL who underwent ASCT by the Center for International Blood and Marrow Transplant Research, PFS and OS at 3 years were 34% and 61%, respectively [21]. These studies support that ASCT resulted in deep responses and played a role in relatively prolonged remission. However, early relapse after transplantation is a major hurdle to overcome to improve survival in pPCL. Recently, results of a prospective study to evaluate the efficacy of an alternative regimen that combined induction therapy with bortezomib and ASCT followed by allogeneic SCT or bortezomib/lenalidomide maintenance was reported [11]. There 40 patients with pPCL received alternative induction therapy with PAD/VCD; the ORR was 69%, including 10% CR and 26% VGPR. After transplantation, 92% of patients achieved more than PR. The median PFS and OS were 15.1 and 36.3 months, respectively. While 35% of patients experienced relapse after allogeneic SCT, only 1 of 7 patients who received maintenance therapy relapsed. Although small in number, lenalidomide maintenance therapy after transplantation could be beneficial in reducing early relapse and improving the duration of remission in pPCL. Thus, rapid reduction of tumor burden with novel agents and ASCT followed by maintenance therapy may be a successful treatment strategy in patients with pPCL.

In the present study, elevated serum LDH and low serum albumin level were significantly associated with poor OS in pPCL. Additionally, patients who did not achieve a complete response after front-line treatment had a very poor prognosis. These risk factors are known to be associated with prognosis in newly diagnosed MM [22]. Beyond these risk factors, cytogenetic abnormalities are important predictors for MM, and a few studies have investigated the prognostic role of cytogenetic abnormalities in pPCL. Because most pPCL studies were based on a small number of patients and were retrospective in nature, the results regarding the prognostic impact of cytogenetic abnormalities differed in each study. In some retrospective studies, del(13q), del(17p), del(1p), ampl(1q), and a complex karyotype were associated with reduced OS, and t(11;14) was associated with favorable outcomes [9, 23]. A recent prospective study, found no prognostic value of abnormalities on FISH or conventional cytogenetic analyses [11]. In this study, cytogenetic abnormalities also showed no prognostic value. However, with the exception of patients who received conventional chemotherapy with much shorter survival outcomes compared with other patient groups, del17p and complex karyotype showed trends towards associated with poor PFS and OS. Thus, larger prospective studies with uniform treatment are needed to further examine the prognostic role of cytogenetic abnormalities in pPCL. Additionally, gene-expression profiling and whole-genome sequencing may be helpful in analyzing and identifying a new prognosis model in pPCL.

In conclusion, induction therapy with novel agents decreased the EM rate and increased the response rates in patients with pPCL. Additionally, performance of ASCT resulted in deep responses and had a role in relatively prolonged remission in pPCL patients who were eligible for ASCT. However, remission duration of pPCL was still shorter than in MM. Consolidation/maintenance treatment with novel agents may be helpful in improving the survival. Additionally, larger prospective studies are needed to establish the optimal treatment strategy and to identify prognostic factors in pPCL.

## MATERIALS AND METHODS

We collected the data retrospectively on patients with pPCL diagnosed at the participating centers of the Korea Multiple Myeloma Working Party between February 1998 and December 2015. The diagnostic criteria for PCL are the presence of > 2 × 10^9^/L peripheral blood plasma cells or plasmacytosis accounting of > 20% in a differential white blood cell count [4]. In this study, we excluded secondary PCL that developed from relapsed or refractory MM. To investigate the clinical characteristics at diagnosis, data for patients with pPCL who were treated or not treated were collected. Treatment regimens, response rates, and survival outcomes were analyzed in patients who were treated. Clinical staging was performed using the ISS. Cytogenetic abnormalities were assessed by conventional cytogenetic studies or fluorescent *in situ* hybridization (FISH). Treatment response was assessed according to the International Myeloma Working Group criteria [4]. ORR was defined as PR or better. This study was approved by the Institutional Review Board of each participating hospital in accordance with the Declaration of Helsinki.

### Statistical analysis

Discrete and continuous variables were evaluated using Fisher’s exact test and the Mann–Whitney *U*–test, respectively. Progression-free survival (PFS) was calculated from the start of treatment to disease progression, or deaths resulting from any causes other than disease progression censored. OS was defined as the period from the date of diagnosis to the date of the last follow-up or death from any cause. PFS and OS were evaluated using Kaplan-Meier estimates and compared using the log-rank test. The estimate of the relative risk of event and its 95% confidence interval (CI) were estimated using the Cox proportional hazard model. Covariates having a *P*-value < 0.1 in the univariate analyses were included in a Cox proportional hazards regression model. All statistical computations were performed using SPSS software (ver. 21; SPSS Inc., Chicago, IL, USA). A *P*-value < 0.05 was considered to indicate statistical significance in all analyses.
